# Unveiling the formation mechanism of characteristic components in steam pot chicken soup with *sanchi-ginseng* based on HPLC, GC–MS combined with metabolomics

**DOI:** 10.1016/j.fochx.2025.102933

**Published:** 2025-08-26

**Authors:** Yanfei Du, Yuan Yang, Guiying Wang, Nannan Zhou, Wen Xun, Chunfang Yang, Shuai Tang, Jiayan Tan, Guozhou Liao

**Affiliations:** a College of Food Science and Technology, Yunnan Agricultural University, Kunming 650201, China; b Livestock Product Processing and Engineering Technology Research Center of Yunnan Province, Yunnan Agricultural University, Kunming 650201, China

**Keywords:** Steam pot chicken, Saponin, Free fatty acid, Small molecule metabolites

## Abstract

Steam pot chicken soup with *sanchi-ginseng* is a traditional Chinese specialty food, and its flavor formation mechanism is currently unclear. The differences of saponins, free fatty acids and small molecular metabolites in the steam pot chicken soup with sanchi-ginseng (PC), steam pot sanchi-ginseng soup (P) and steam pot chicken soup (C) groups were analyzed by HPLC, GC–MS and UHPLC-QE-MS, respectively. The results showed that the concentration of three saponins in the PC group was significantly reduced compared to the P group. The FFA concentration in PC group was significantly higher than P and C groups. 13 differential metabolites shared in common were identified in the two comparison groups. KEGG results indicated that amino acid and fatty acid metabolism were the major metabolic pathways affecting the differences in steam pot chicken soup with sanchi-ginseng. This study elucidate the formation mechanism of the flavor characteristics of steam pot chicken soup with *sanchi-ginseng.*

## Highlights

• The content of three saponins in the PC group was significantly reduced compared to the P group.
• The FFA content in the PC group was significantly higher than that in the P and C groups.
• Palmitic acid (C16:0) and stearic acid (C18:0) were the major FFA in PC group.
• A total of 15 key differential compounds metabolites were identified between PC and P groups.
• A total of 7 key differential compounds metabolites were detected between PC and C groups.

## 1 Introduction

*Sanchi-ginseng* (roots of *Panax notoginseng*) is a perennial herb in the Araliaceae family. Due to its hemostatic and blood activating properties, it is widely used in some East Asian countries to treat organ damage and cardiovascular diseases (Ng, 2006). Especially in China, the medicinal history of *sanchi-ginseng* can be traced back to the Ming Dynasty in the 16th century. *Sanchi-ginseng,* as the main raw material for producing Yunnan Baiyao, is widely planted and distributed in several provinces in southwest China, such as Yunnan and Guangxi provinces (Xu et al., 2019). *Panax notosaponins* (PNS) is a chemical component derived from Ginseng saponins and contains various monomers, such as *Panax notosaponins* R1, ginsenosides Rg1, and ginsenosides Re (Liu et al., 2010). PNS has anti-inflammatory, antioxidant, and protective effects on vascular endothelial cells and is the key active ingredient in Xuesaitong and other proprietary Chinese medicines (Guo et al., 2019; Zhang et al., 2019), which are best-selling prescriptions for the treatment of cardiovascular and cerebrovascular diseases in China (Li et al., 2020).

Wuding Chicken (the first of the six famous chickens in Yunnan) is a famous local fine chicken breed in China, originating from Wuding County, Chuxiong Yi Autonomous Prefecture, Yunnan Province. Wuding chicken is characterized by its large size, meaty, tasty meat, and rich nutrition (Jia et al., 2024). Chicken is recognized as a significant dietary source of high-quality protein.Its global utilization is facilitated by economical pricing, low fat and cholesterol content, and broad cultural and religious acceptability (Cao et al., 2021). Chicken soup is a stew made of chicken as raw material. Because of its rich nutrition and unique flavor, it is favored by consumers all over the world and has become a popular food (Qi et al., 2023). In traditional Chinese food culture, chicken soup is regarded as a tonic with a therapeutic effect because it can prevent colds, relieve inflammation, and improve immunity, especially suitable for children, the elderly, and the infirm (Guan et al., 2023).

Medicinal diets are widely accepted as a kind of health food therapy, and steam pot chicken soup with *sanchi-ginseng* is a kind of medicinal diet that is both delicious and disease-preventing(Guo et al., 2019; Li et al., 2020). During the steaming process, free fatty acids in chicken meat and saponins in Panax notoginseng, among other active ingredients, are extracted into the chicken broth by the action of steam, creating a unique flavor (Xiao et al., 2018; Toh, New, Koh., 2009; Jia et al., 2024). The cooking mechanism derives from the synergy between the physical structure of its special purple sand steam pot and the thermodynamic process of steam. When the steam comes into contact with the low-temperature surface of ingredients, it undergoes phase change and condensation, releasing latent heat and forming high-purity distillate. This liquid phase fully integrates with chicken-derived substances, resulting in a colloidal soup base characterized by low osmotic pressure and high amino acid concentration (Lian, Cheng, & Sun, 2023; Lian, Cheng, Wang, & Sun, 2023). The porous nature of the clay material provides a progressive thermal buffer effect, ensuring that chicken and other ingredients are cooked gradually within the appropriate temperature range. This helps maximize the preservation of muscle fiber structural integrity and the retention of flavor-presenting substances (Duan, Liang, Huang, Zhang, Sun, & Li, 2021; Luo, 2020). The stability of *Panax ginseng* saponin compounds (such as ginsenosides Rg1 and Rb1) is guaranteed under the sub-boiling steam environment (Zhang et al., 2021), forming a synergistic medicinal-food complex system.

As a traditional Yunnan specialty food, steam pot chicken soup with *sanchi-ginseng* is popular among consumers for its unique cooking method, delicious taste, and tonic effect. However, most of the current research reports focus on the quality differences between chicken breeds or chicken parts, as well as the effects of various processing methods on the quality and flavor of chicken soup (Feng et al., 2018), whereas fewer studies have been reported on the formation mechanism of the quality characteristics of steam pot chicken soup with *sanchi-ginseng*, and the characteristic components of it is not yet clear, which to some extent restricts the research and promotion of steam pot chicken soup with *sanchi-ginseng* . In this study, the differences of saponins, free fatty acids (FFA), and small molecule metabolites in steam pot chicken soup with *sanchi-ginseng* (PC), steam pot *sanchi-ginseng* soup (P), and *s*team pot chicken soup (C) groups were analyzed by high-performance liquid chromatography (HPLC), gas chromatography–mass spectrometry (GC–MS), and Ultra-performance liquid chromatography-quadrupole-electrostatic field Orbitrap - mass spectrometry (UHPLC-QE-MS), respectively. Through KEGG analysis, the main metabolic pathways affecting the differences in steam pot chicken soup with *sanchi-ginseng* were identified, revealing the important mechanism by which the addition of *sanchi-ginseng* affects the flavor of chicken soup, helping to promote the development and utilization of Wuding chicken and *Sanchi-Ginseng* resources, as well as the modernization of traditional cuisine.

## 2 Materials and methods

### 2.1 Materials and chemicals

300-day-old Wuding chickens (castrated roosters) uniformly slaughtered by Wuding Zhenji Agricultural Technology Development Co.,Ltd. (Yunnan, China), packaged in sterile sealed bags, and transported to the laboratory at 0–4 °C. *Sanchi-ginseng* (roots of *Panax notoginseng*) was provided by Wenshan Huaxin Sanqi Technology Co., Ltd. in Yunnan Province, China. Notoginsenoside R1 (HPLC ≥98 %), ginsenoside Rg1 (HPLC ≥98 %), and ginsenoside Rb1 (HPLC ≥98 %) were purchased from Shanghai yuanye Bio-Technology Co., Ltd. 49 mixed fatty acid methyl esters (GC standard) were purchased from Sigma-Aldrich (Shanghai) Trading Co., Ltd. The steam pots (20 cm diameter) were purchased from Jianshui Yinghong Purple Pottery Boiler Factory in Yunnan Province, China.

### 2.2 Sample preparation

According to the optimal processing parameters determined by our team's previous pre-experiment, the samples were processed based on 100 g of chicken breast meat(cut the chicken meat into small pieces measuring 1 cm × 1 cm × 1 cm), with a 2 % addition of *sanchi-ginseng* and 2 % salt, and a steaming time of 2 h(the amount of salt and *sanchi-ginseng* added is calculated based on the weight of the chicken meat).This experiment included three treatment groups, namely the steam pot chicken soup with *sanchi-ginseng* (PC) group (chicken 100 g, *sanchi-ginseng* 2 %, salt 2 %), the steam pot *sanchi-ginseng* soup (P) group (*sanchi-ginseng* 2 %, salt 2 %), and the steam pot chicken soup (C) group (chicken 100 g, salt 2 %). The raw materials were placed in the Jianshui purple pottery pot and steam treated for 2 h. After cooking, use a strainer to remove the chicken and *sanchi-ginseng*, cool to 50–60 °C, then slowly pour the chicken soup into the pre-prepared gauze filter device. Repeat the process until the soup is free of visible impurities and stored at −20 °C for testing.

### 2.3 Determination of saponin concentration

0.3 g of freeze-dried PC and P group sample powders were placed in a 50 mL centrifuge tube, accurately added with 25 mL of methanol, and allowed to stand overnight. The mixture was thoroughly shaken, sonicated for 40 min, cooled to room temperature, and then weighed. Finally, 25 mL of methanol were added, shaken well, and filtered through a microporous membrane (0.45 μm) to obtain the test solution. R1, Rg1, and Rb1 standards were diluted with methanol to prepare control samples at different concentrations (25 mg/L, 50 mg/L, 100 mg/L, 200 mg/L, 400 mg/L) for HPLC analysis. The chromatographic conditions were as follows: C18 column (4.6 mm × 250 mm, 4.6 μm); mobile phase: water (A)-methanol (B); gradient elution conditions: 0–6 min, 30 % B; 6–14 min, 40 % B; 14–20 min, 30 % B; 20–30 min, 20 %. The flow rate was 1.0 mL/min, and the column temperature was 30 °C. Injection volume: 10 μL; detection wavelength: 203 nm.

### 2.4 Determination of free fatty acids

GC–MS analysis was performed using an Agilent 7890B gas chromatograph system coupled with a Agilent 5977B mass spectrometer. The analysis of free fatty acids was based on the previous method of our research group (Liu et al., 2019), with slight modifications. 50 μL of the sample was placed in a 2 mL EP tube, and 450 μL of extraction solution (isopropanol: n-hexane = 2:3 (*V*/V), containing 0.2 mg/L internal standard) was added. Steel balls were added, and the mixture was stirred for 30 s. Then, the mixture was sonicated in an ice water bath for 10 min. The sample was centrifuged at 4 °C and 12,000 rpm for 15 min, and the supernatant was taken out and placed in a new 1.5 mL EP tube. Next, 500 μL of extraction solution was placed in the remaining sample, and the above steps were repeated to combine the two supernatants again. After vortexing for 10 s, 800 μL of the combined supernatant was taken out, dried with nitrogen, and 500 uL of methanol solution and 1000 μL of trimethylsilyldiazomethane solution were added. The mixture was left at room temperature for 30 min and dried with nitrogen. Finally, 160 μL of n-hexane was added and centrifuged at 12000 rpm for 1 min. The supernatant was then placed in an automatic sample bottle for measurement. The specific parameters were as follows: the sample was injected in a 1 μL injection volume through the split-flow mode (split ratio of 5:1), the flow rate of spacer purge was 3 mL/min, helium was used as the carrier gas, and the column was chosen as a DB-FastFAME capillary column (90 m × 250 μm × 0.25 μm), with the column pressure at a constant level of 46 psi. The column incubator used a multi-stage programmed warming: 50 °C，1 min；50 °C/min-200 °C，15 min；2 °C/min-210 °C，1 min；10 °C/min-230 °C，15 min. The temperature of the front sample port and the transfer line was set at a constant temperature of 46 psi. The temperature of the forward sample port and the transfer line was set at 240 °C, the temperature of the ion source at 230 °C, the temperature of the quadrupole at 150 °C, and the ionization voltage at −70 eV. The mass spectrometry was performed in the Scan/SIM mode with a mass scanning range of 33–400 *m*/*z*, and a solvent delay of 7 min was set to prevent the solvent peaks from interfering with the mass spectrometry.

### 2.5 Analysis of small molecule metabolites

Small molecule metabolites were analyzed based on our previous research methods (Yang et al., 2022) with slight modifications. 100 μL of the chicken soup sample was placed in an EP tube, and 400 μL of extraction solution (methanol: acetonitrile = 1:1, *V*/V) and isotopic labeled internal standard mixture were added. After vortex mixing for 30 s, the sample was treated with ultrasound in an ice bath for 10 min, and then the mixture was left to stand at −40 °C for 1 h. Subsequently, the sample was centrifuged at 4 °C and 12,000 rpm for 15 min, and the supernatant was taken and placed in a sample bottle for machine testing. The key parameters were set as follows: the sheath gas flow rate and auxiliary gas flow rate were set at 50 arb and 15 arb, respectively, and the capillary temperature was maintained at 320 °C to ensure efficient desolventization. The mass spectrometry acquisition was carried out in positive and negative ion modes, and the spray voltages were set to +3.8 kV (positive ion mode) and − 3.4 kV (negative ion mode), respectively.The resolution of full MS was set to 60,000, and the resolution of the MS/MS secondary fragmentation was increased to 15,000, and the collision energy was set to 20 %, 30 %, and 40 % using the stepped normalized collision energy (NCE) mode. The collision energy is set to 20 %, 30 %, and 40 % for multi-stage fragmentation. (the metabolomics testing for this experiment was provided by Shanghai Baiqu Biomedical Technology Co., Ltd.)

### 2.6 Statistical analysis

Each experiment was repeated six times, and all data were expressed as mean ± standard deviation. All data were initially sorted using Excel 2010, analyzed for variance and Duncan's multiple comparisons using SPSS 19.0 software, and subjected to multivariate statistical analysis using the MetaboAnalyst 6.0 platform (Chong et al., 2019). The significance level was considered as *P* < 0.05.

## 3 Results and discussion

### 3.1 Quantitative analysis of saponins

**Fig. 1:**
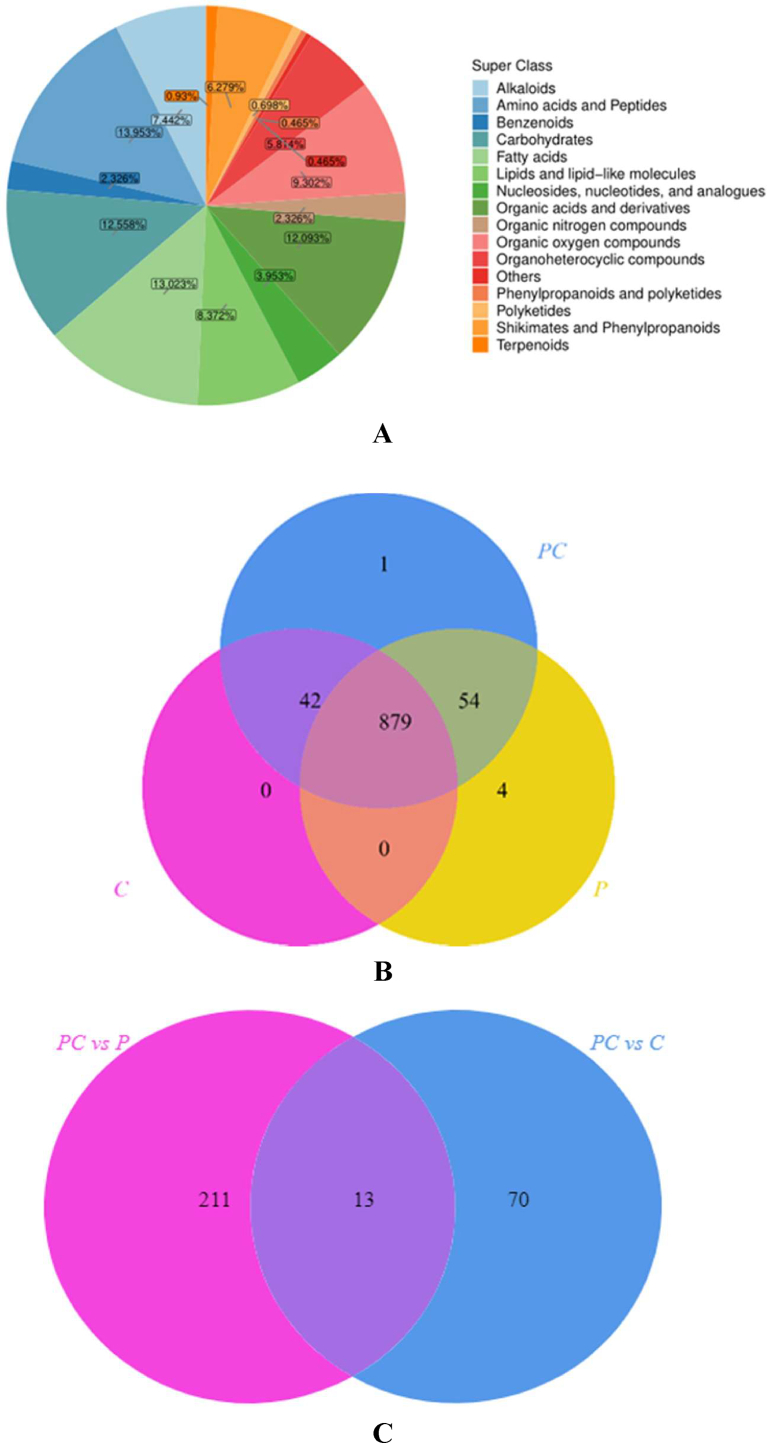
Qualitative analysis of small molecule metabolites in different groups of soup. Classification pie chart of small molecule metabolites (A), Venn diagram of small molecule metabolites in different groups of soup (B), and Venn diagram of differential metabolites in the two comparison groups (C).

The HPLC profiles of notoginsenoside R1, ginsenoside Rg1, and ginsenoside Rb1 in PC and P groups are shown in Fig. 1S. Excessive steaming time or high steaming temperature can significantly reduce the concentration of major saponin components (Toh et al., 2009). As shown in Table S1, the concentration of R1, Rg1, and Rb1 in the PC group were 19.52 mg/L, 61.04 mg/L, and 20.74 mg/L, respectively. The concentration of R1, Rg1, and Rb1 in the P group were 24.90 mg/L, 95.12 mg/L, and 35.13 mg/L, respectively. The concentrations of R1, Rg1, and Rb1 in the P group were 27.56 % (*P* < 0.05), 55.83 % (*P* < 0.05), and 69.38 % (*P* < 0.05) higher than those in the PC group, respectively. During the cooking process, heat treatment can degrade saponins, leading to a decrease in the concentration of major saponins (Xiong et al., 2019). Under the same process conditions, the concentration of three saponins in the PC group was lower than that in the P group, which may be due to the chemical reaction between the saponins in the PC group and a certain component in the chicken meat during heat treatment, resulting in a decrease in saponin concentration (Wang et al., 2012; Wang et al., 2020).

### 3.2 Analysis of free fatty acid

#### 3.2.1 Compositional analysis of free fatty acids

During the cooking process, meat products undergo fat degradation due to thermal reactions, resulting in the release of some fatty acids. These fatty acids are not only precursors of volatile flavor compounds, but also important taste-active substances (Madruga et al., 2010). As shown in Fig. S2, 21 kinds of free fatty acids were ultimately detected in this study through GC–MS analysis, including caprylic acid (C8:0), capric acid (C10:0), lauric acid (C12:0), tridecanoic acid (C13:0), myristate acid (C14:1), pentadecanoic acid (C15:0), palmitic acid (C16:0), palmitoleic acid (C16:1), heptadecanoic acid (C17:0), and heptadecane. Oleic acid (C17:1), stearic acid (C18:0), oleic acid (C18:1), isooleic acid (C18:1), linoleic acid (C18:2/LA), α-linolenic acid (C18:3/ALA), arachidonic acid (C20:0), eicosenic acid (C20:1), arachidonic acid (C20:4/AA), behenic acid (C22:0), and erucic acid (C22:1). Our previous research had found that palmitic acid, stearic acid, oleic acid (C18:1n-9), and linoleic acid (C18:2n-6) were the main fatty acids of Wuding chickens (Xiao et al., 2018), which is similar to this study. The different fatty acid composition is the main reason for the different flavors of various meat products. For example, the main fatty acids in chicken meat are palmitic acid, stearic acid, oleic acid, and linoleic acid, while the main fatty acids in pork are palmitic acid, stearic acid, oleic acid, and linolenic acid (Barola et al., 2020; Xiao et al., 2018). However, the lowest concentration of fatty acids in dry-cured ham is palmitic acids (Li et al., 2018). The most abundant free fatty acids found in dairy products such as milk are 7-hydroxystearic acid and 10-hydroxystearic acid (Sun et al., 2018).

#### 3.2.2 Differential analysis of free fatty acid concentration

Li et al. (2022) pointed out in previous studies that important components affecting the quality of chicken meat include organic acids and their derivatives, lipids and lipid molecules, nucleotides and analogues. Research has found that cinnamaldehyde inhibits the degradation of methionine-containing peptides and promotes the degradation of aspartic acid and peptides in beef broth (Li et al., 2020).Table 1 reveals that the composition of free fatty acids in the PC group and C group is identical, with a total of 21 types of free fatty acids, while there are 18 types in the P group. Compared with the PC and C groups, the P group did not contain palmitoleic acid (C16:1), eicosanoic acid (C20:1), and arachidonic acid (C20:4/AA). The concentration of saturated fatty acids, monounsaturated fatty acids, and polyunsaturated fatty acids in the PC group was the highest, at 64.22 μg/mL, 37.99 μg/mL, and 11.56 μg/mL, respectively. The concentration of palmitic acid (C16:0) and stearic acid (C18:0) was also highest in PC group, which is consistent with the results found in other studies (Nkukwana et al., 2013). Palmitic acid and stearic acid are excellent sources of bioactive lipids (Salazar et al., 2020). Simultaneously, aroma precursors such as medium-long chain free fatty acids (C > 6) can undergo further degradation as substrates, resulting in the production of small molecular volatile flavor substances like aldehydes and acids (Huang et al., 2020), thereby enhancing food flavor.

**Table. 1:** The concentration and composition of free fatty acids in different groups of soups (μg/mL).

Free fatty acid	PC	P	C
Octanoic acid(C8:0)	0.08 ± 0.01^a^	0.02 ± 0.01^b^	0.02 ± 0.01^b^
Decanoic acid(C10:0)	0.13 ± 0.03^a^	0.04 ± 0.02^b^	0.04 ± 0.02^b^
Lauric acid(C12:0)	0.12 ± 0.02^a^	0.06 ± 0.02^b^	0.06 ± 0.02^b^
Tridecanoic acid(C13:0)	0.18 ± 0.07^a^	0.10 ± 0.02^a^	0.08 ± 0.01^a^
Myristic acid(C14:0)	1.13 ± 0.04^a^	1.04 ± 0.09^a^	0.99 ± 0.10^a^
Myristoleic acid(C14:1)	1.06 ± 0.07^a^	0.62 ± 0.03^b^	0.44 ± 0.03^c^
Pentadecanoic acid(C15:0)	1.48 ± 0.07^a^	0.67 ± 0.03^b^	0.65 ± 0.03^b^
Palmitic acid(C16:0)	34.57 ± 0.86^a^	5.55 ± 0.63^c^	28.69 ± 1.80^b^
Palmitoleic acid(C16:1)	1.33 ± 0.03^a^	ND	1.29 ± 0.06^b^
Heptadecanoic acid(C17:0)	1.56 ± 0.20^a^	0.83 ± 0.02^b^	0.89 ± 0.06^b^
heptadecenoic acid(C17:1)	16.99 ± 2.73^a^	5.30 ± 1.07^c^	11.56 ± 1.51^b^
Stearate(C18:0)	23.13 ± 2.24^a^	6.83 ± 0.27^c^	11.69 ± 1.45^b^
Oleate(C18:1)	14.66 ± 3.28^a^	2.05 ± 0.23^b^	11.68 ± 1.17^a^
Isoleic acid(C18:1)	2.28 ± 0.18^a^	0.71 ± 0.07^c^	1.53 ± 0.29^b^
Linoleic acid(C18:2/LA)	8.23 ± 0.20^a^	1.80 ± 0.16^c^	6.50 ± 0.64^b^
Alpha-linolenic acid(C18:3/ALA)	2.32 ± 0.21^a^	0.92 ± 0.03^c^	1.42 ± 0.18^b^

Note: Different letters on the shoulder of the same row indicate a significant difference (*P* < 0.05).

PC represents the group of steam pot chicken soup with *sanchi-ginseng*, P represents the group of steam pot *sanchi-ginseng* soup, and C represents the group of steam pot chicken soup.

### 3.3 Analysis of small molecule metabolites

#### 3.3.1 Qualitative analysis.

Several metabolic pathways can interact to form the small and medium molecule metabolites in chicken soup. 525 and 455 small molecule metabolites were detected by UHPLC-QE-MS in both positive and negative ion modes. Fig. 1A primarily displays small molecular metabolites, including alkaloids, amino acids, peptides, carbohydrates, fatty acids, lipids, lipid molecules, nucleosides, nucleotides, and their analogues. Fig. 1B shows the Venn diagram of small molecule metabolites in the three groups. As shown in Fig. 1B, there were 933 small molecule metabolites in the PC and P groups, 921 small molecule metabolites in the PC and C groups, and 879 small molecule metabolites in the P and C groups. In addition, 879 small molecule metabolites were detected in the PC group, which were common among the three groups. Panthiethylamine was a unique small molecule compound in the PC group, while acetylcholine, adenosine dialdehyde, 8-amino-7-oxononaic acid, and N4- (acetyl-beta-d-glucosamine) asparagine were the four unique small molecules in the P group.

#### 3.3.2 Multivariate statistical analysis

**Fig. 2:**
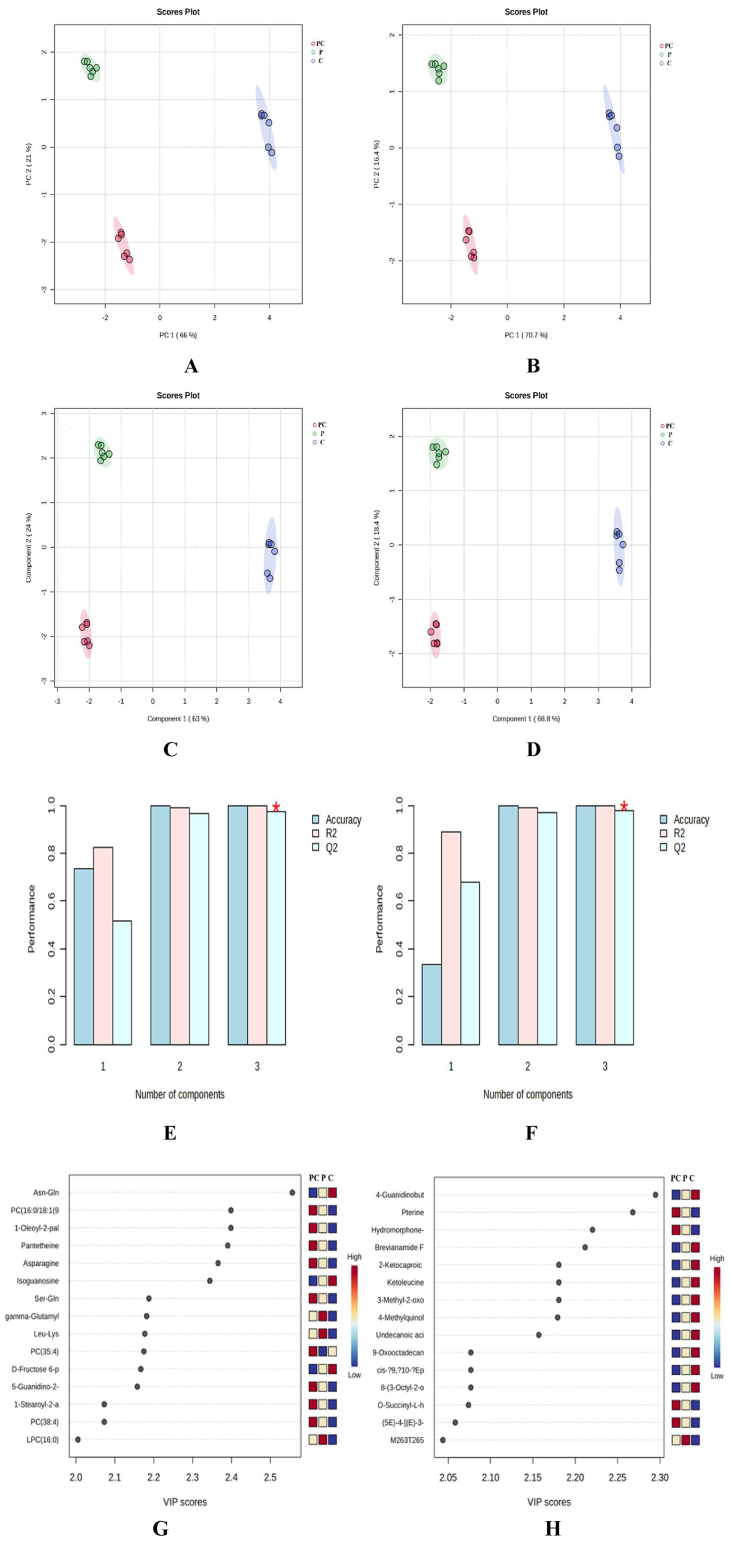
Statistical analysis of small molecule metabolites in different groups of soup. PCA score plots and OPLS-DA score plots of all experimental samples in ESI+ (A, C) and ESI- mode (B, D), cross-validation results of PLS-DA models of ESI+ (E) and ESI- (F) mode, and VIP score diagram in ESI+ (G) and ESI- (H) mode.

As shown in Fig. 2A and 2B, there was a clear separation trend between the groups in both positive and negative ion modes, indicating that the small molecule metabolites in the three groups were different. PLS-DA analysis can make complex data easier to understand and provide a clearer view of high-dimensional data. When there is not much difference between sample groups, it also makes it easier to identify possible metabolites related to metabolic changes (Chen et al., 2023). In addition, PLS-DA analysis can effectively detect differences between groups (Xue et al., 2023). In this study, samples from different groups were analyzed using the PLS-DA model to further differentiate their differences and obtain reliable differences between small molecule metabolites. As shown in Fig. 2C and 2D, samples from different groups were effectively separated under different ion modes, revealing differences in small molecule metabolites among the three groups. The cross-validation results of PLS-DA (Fig. 2E and 2F) showed that in both positive and negative ion modes, the evaluation parameters R^2^ and Q^2^ of PLS-DA model were close to 1, indicating the effectiveness and high prediction ability of this model. As shown in Fig. 2G and 2H, the top 15 differentially expressed metabolites with VIP values were screened out among the three treatment groups. The higher the VIP value, the greater the contribution to the model's ability to distinguish sample differences, which plays a crucial role in sample differentiation and is a clear indicator of differences. Table S2 shows the 15 different metabolites with the highest VIP values among the three treatment groups, which were discovered using PubChem, HMDB, and KEGG compound databases in both positive and negative ion modes.

#### 3.3.3 Differential compound analysis

The PCA score chart (Fig. S4) clearly shows a separation trend between samples in the positive and negative ion modes, suggesting a difference between small molecular metabolites in the PCvs P group and the PCvs C group. While PCA, an unsupervised dimension reduction method, is insensitive to variables with little correlation, PLS-DA effectively solves this problem. On the other hand, orthogonal partial least square discriminant analysis, a supervised statistical method of discriminant analysis, combines orthogonal signals and PLS-DA to screen differential variables. Partial least squares regression established the relationship model between metabolite expression and sample class, predicting the sample class. The PC vs P group and PC vs C group had better data separation, as shown by the OPLS-DA score chart (Fig. S5). There were also clear differences in the samples' small and medium-sized molecular metabolites. We used permutation detection to verify the accuracy of this model. The OPLS-DA verification diagram (Fig. S3) reveals that the PCvsP group and PCvsC group exhibit superior interpretation rates and prediction abilities in the two models.

**Fig. 3:**
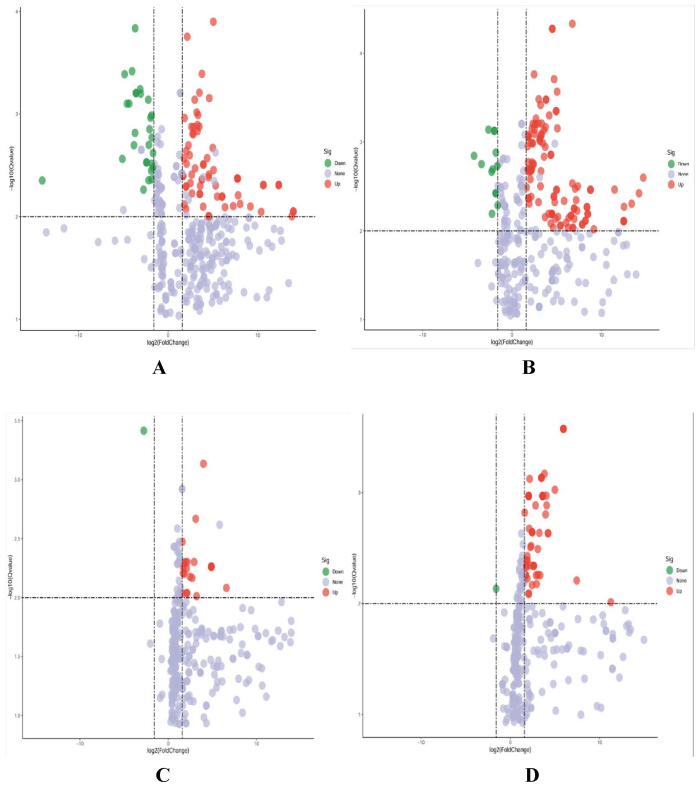
Analysis of differential metabolites between different groups of soup. Volcano plot of PC vs. P group in ESI+ (A) and ESI- (B) mode, Volcano plot of PC vs. C group in ESI+ (C) and ESI- (D) mode.

Based on the OPLS-DA model, this study looked at small and medium-sized molecules in the PCvs P and PCvsC groups. Metabolites were screened with VIP ≥ 1.2, FC ≥ 3, or FC ≤ 1/3 and *P* < 0.01. The volcano map (Fig. 3) revealed the identification of a total of 372 small molecule metabolites in the PC vs. P group under the positive ion mode, with 94 different metabolites among them. In comparison to group P, there was an upregulation of 66 different metabolites, primarily valine and tyrosine. The expression of 28 different metabolites, mainly N4-(acetyl-beta-d- glucosamine) asparagine, was down-regulated. The PCvs P group identified a total of 345 small molecule metabolites in the negative ion mode, of which 130 were differential metabolites. If you compare the PC group to the P group, 117 differential metabolites dominated by 5-methylcytosine went up, while 13 differential metabolites dominated by ginsenoside Rg3 and ginsenoside RG3 went down. The PC vs.C group identified a total of 334 small molecule metabolites, of which 23 showed differential expression, 22 showed upregulation, and 1 showed downregulation. Group PC significantly upregulated 22 small molecule metabolites, mainly L-urea-alanine, compared to group C. Asparagine glutamine was significantly down-regulated in the PC group. The PCvs C group identified 332 small molecule metabolites in the negative ion mode, with 60 showing differential expression, 59 showing up-regulation, and 1 showing down-regulation. Group PC significantly upregulated 59 small molecule metabolites, mainly 2-hydroxycinnamic acid, compared to group C. Undecanoic acid is a significantly down-regulated substance in the PC group.

#### 3.3.4 Pathway analysis of differential metabolites

**Fig. 4:**
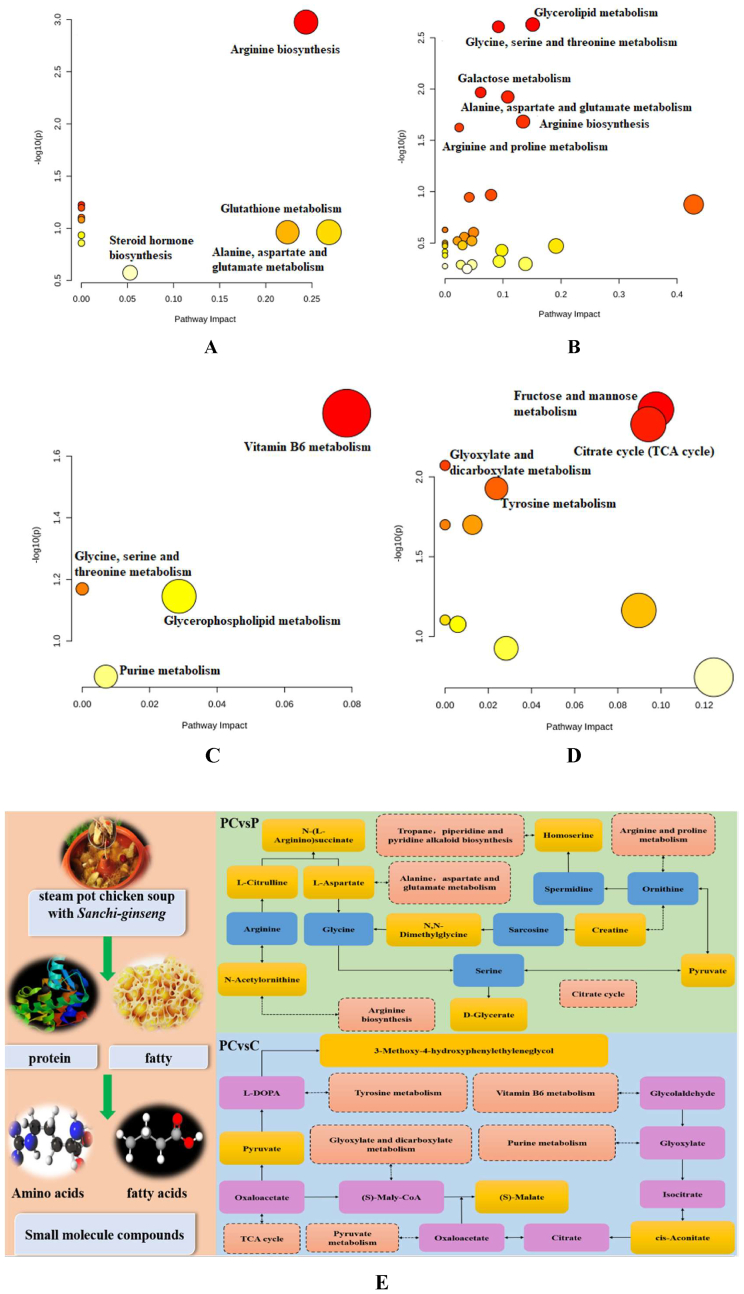
KEGG-based metabolic pathway analysis. Pathway enrichment analysis of differential compounds in PC vs. P group in ESI+ (A) and ESI- (B) mode, pathway enrichment analysis of differential compounds in PC vs. C group in ESI+ (C) and ESI- (D) mode, the metabolic pathway network of steam pot chicken soup with *sanchi-ginseng* (E), blue and purple represents the metabolites identified in this study, and yellow represents the key metabolites identified.

Fig. 1C shows that we screened 224 and 83 different metabolites in the positive and negative ion modes for group PC vs. P and group PC vs. C, respectively, and found 13 common metabolites. These different metabolites may play an important role in group PC, group P, and group C and affect their metabolism. We performed pathway enrichment based on the screened differential metabolites. Fig. 4 illustrates the metabolic pathway enrichment analysis of the group PC vs. P and the group PC vs. C in both positive and negative ion modes. The Fig. reveals that group PC vs P under positive ion mode has 10 metabolic pathways, including glutathione metabolism, arginine biosynthesis, alanine, and steroid hormone biosynthesis. There were four metabolic pathways in the PC vs. C group, namely vitamin B6 metabolism, purine metabolism, glycine, serine, and threonine metabolism, and glycerol phospholipid metabolism. (Yang et al., 2022) reported that triglyceride metabolism, adipokine signaling, linoleic acid, glycerolipids, and other lipid metabolic pathways could lead to changes in chicken soup flavor. Based on the impact value and the *P* value (impact >0.01, *P* < 0.05), a key metabolic pathway was selected for arginine biosynthesis in the PC vs. P group. We mapped l-aspartate and L-citrulline as key metabolites through KEGG metabolite data mapping. The PC vs C group identified vitamin B6 metabolism as a key metabolic pathway, and KEGG metabolite data mapping identified pyridoxine as the key compound. Pyridoxine, a form of vitamin B6, readily transforms into pyridoxal 5-phosphate (PLP), a crucial cofactor in enzymatic reactions (Kall, 2003; Wilson et al., 2019).There were 29 metabolic pathways in group PC vs. P and 11 metabolic pathways in group PC vs. C under negative ion mode. For the PC vs. P group, six important metabolic pathways were found based on the impact value (impact >0.01, *P* < 0.05). These were triglyceride metabolism, galactose metabolism, alanine, aspartate, and glutamate metabolism, arginine biosynthesis, and arginine and proline metabolism. The mapping of KEGG metabolite data revealed a total of 13 key metabolites, including glycerol phosphate, triglyceride, D-glyceride, N, *n*-dimethylglycine, and creatine. Li et al. (2022) also reported in previous studies that the quality of chicken soup is significantly influenced by organic acids and their derivatives, lipids and lipid molecules, nucleosides, nucleotides, and analogs. Among them, pyruvate is involved in three metabolic pathways. Pyruvate is a phenolic acid that exists in plants and has antioxidant, anticancer, and antibacterial activities (Zieniuk, 2023). D-glyceride, creatine, *n*-acetyl-L-aspartic acid, and N-(L-arginine) succinate participate in two metabolic pathways, which aligns with the findings of Fedorov et al. (2020). The PC vs. C group revealed four key metabolic pathways: fructose and mannose metabolism, tricarboxylic acid cycle, glyoxylic acid and dicarboxylic acid metabolism, and tyrosine metabolism. KEGG metabolite data mapped six key metabolites, including d-fructose, D-mannose, (*S*)-malic acid, and cis-mononic acid. Among them, (*S*)-malic acid and (*Z*)-aconitic acid participate in two metabolic pathways. *Cis*-Aconitic acid, which mainly exists in the roots and leaves of beets, sugarcane, and other plants, is one of the important metabolites of the tricarboxylic acid cycle (Chun, Lee & Park, 2020). Additionally, notoginseng boiler chicken broth contains caffeine, ginsenoside Rg3, ginsenoside Rf, ginsenoside Rg2, notoginseng R2, and other physiologically active ingredients. This suggests that adding notoginseng to chicken broth can enhance its active ingredient concentration, thereby enhancing its physiological function, in line with the research findings of Wang et al. (2024).

Based on the KEGG database, the relevant metabolic pathways of the compounds in PC vs. P and PC vs. C were further identified. As shown in Fig. 4E, the concentration of amino acids such as serine, creatine, ornithine, and arginine in PC vs. P were significantly higher in PC group than in P group, and the concentration of amino acids such as oxaloacetic acid, (*S*)-Maly-CoA, (*S*)-Malate, and cis-Aconitate in PC vs. C were significantly higher in PC group than in C group. This suggests that amino acids and fatty acids produced during the degradation of chicken proteins and fats, as well as the addition of *sanchi-ginseng* may largely affect the quality of PC soup. The degradation of chicken protein and fat resulted in more amino acids and fatty acids further entering the citric acid cycle and TCA cycle through pyruvate, affecting other metabolites in the metabolic process. The concentration of small molecule compounds such as Jasmine lactone, 4-Amino-5-hydroxymethyl-2- methylpyrimidine, and melibiose in PC soup was significantly reduced, suggesting that the sensory compounds in PC soup may also be closely related to the metabolism of glycerophospholipids, fructose, and mannose. In addition, some amino acid metabolism and biosynthesis pathways also affect the sensory quality of chicken soup, and studies have shown that the production of organic acids may also originate from amino acid metabolism, lipid oxidation and other pathways during the heating process of chicken soup, thus affecting the overall flavor of the soup (Yue et al., 2025).

## 4 Conclusion

In this study, the formation mechanism of quality characteristic of PC soup was explored using HPLC, GC–MS, and UHPLC-QE-MS. Three saponins were significantly different between the PC and P groups. The PC and C groups had the same FFA composition and the concentration of FFA in PC group was significantly higher than that in P and C groups. The concentration of the three saponins were found to be significantly different between the PC and P groups. Palmitic acid, stearic acid, linoleic acid, and α-linolenic acid were the main free fatty acids in the PC group. A total of 980 metabolites in the chicken soup were detected, while 13 different metabolites were found in the two comparison groups. The study identified 15 metabolites as the key metabolites of PC vs. P, including pyruvate, d-glyceride, creatine, *N*-acetyl-l-aspartate, and N-(L-arginine) succinate. Seven compounds, including pyridoxine, (*S*)-malic acid, (*Z*)-aconite acid, and d-fructose were also identified as key metabolites of PC vs. C. The research results are of great significance for the rational development and utilization of Yunnan's characteristic resources, as well as for promoting the development of the Wuding chicken and *sanchi-ginseng* industries. However, current research has certain limitations.In future research work, it is very necessary to further study the mineral content in steam pot chicken soup with *sanchi-ginseng* and the interaction between saponins and chicken protein to reveal the mechanism behind the formation of chicken soup flavor in greater depth so as to provide a scientific theoretical basis for regulating its quality.

## CRediT authorship contribution statement

**Yanfei Du:** Writing – original draft, Software, Methodology, Formal analysis, Data curation. **Yuan Yang:** Software, Formal analysis, Data curation. **Guiying Wang:** Supervision, Resources, Project administration, Funding acquisition. **Nannan Zhou:** Validation, Software, Data curation. **Wen Xun:** Validation, Software. **Chunfang Yang:** Software, Formal analysis. **Shuai Tang:** Software, Methodology. **Jiayan Tan:** Software, Methodology. **Guozhou Liao:** Writing – review & editing, Supervision, Resources, Project administration, Funding acquisition.

## Declaration of competing interest

The authors declare that they have no known competing financial interests or personal relationships that could have appeared to influence the work reported in this paper.

## Data Availability

I have shared the link to my data/code at the Attach File step
