# Supplementary material for: Unveiling the formation mechanism of characteristic components in steam pot chicken soup with sanchi-ginseng based on HPLC, GC–MS combined with metabolomics
Source: Food Chem X. 2025 Aug 26;30:102933. doi: 10.1016/j.fochx.2025.102933 (PMC12445720; doi:10.1016/j.fochx.2025.102933)
Supplement: Supplementary material 3 [file mmc3.docx]

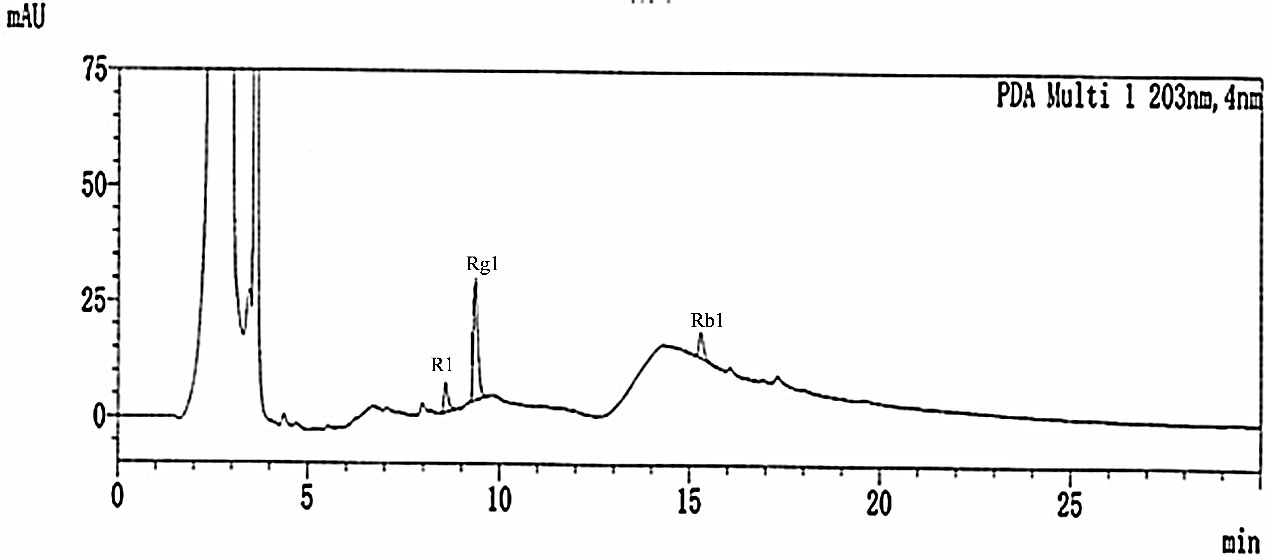


A


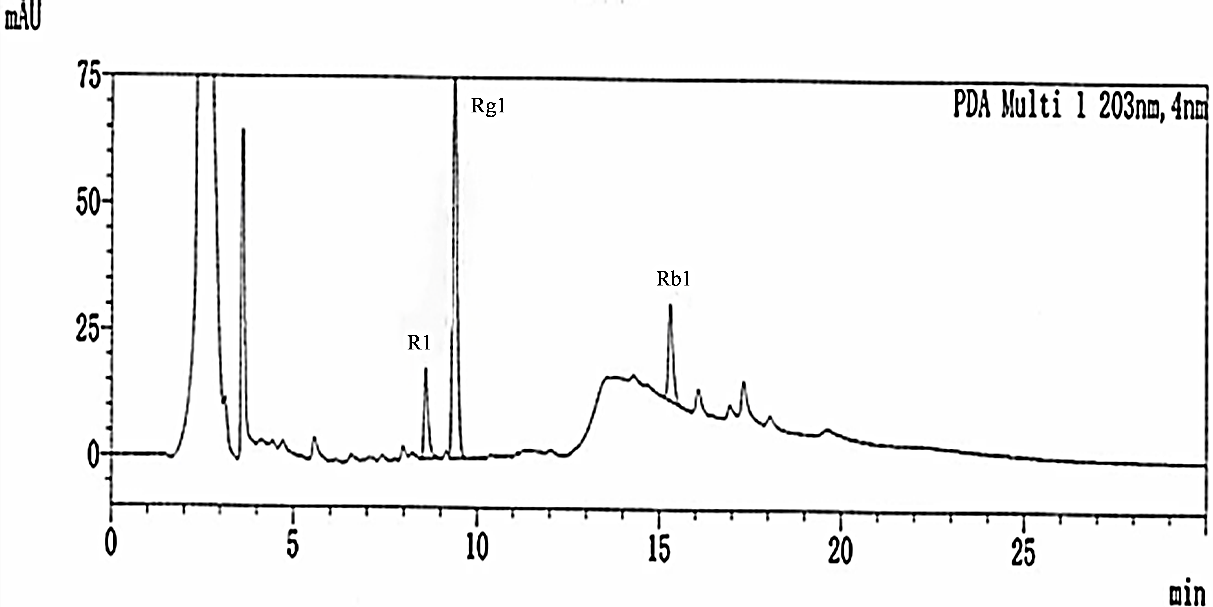


B

**Fig. S1 HPLC chromatograms of three types of saponins.** Three saponins in PC group (A), three saponins in P group (B). PC represents the group of steam pot chicken soup with *sanchi*-*ginseng*, P represents the group of steam pot *sanchi-ginseng* soup.


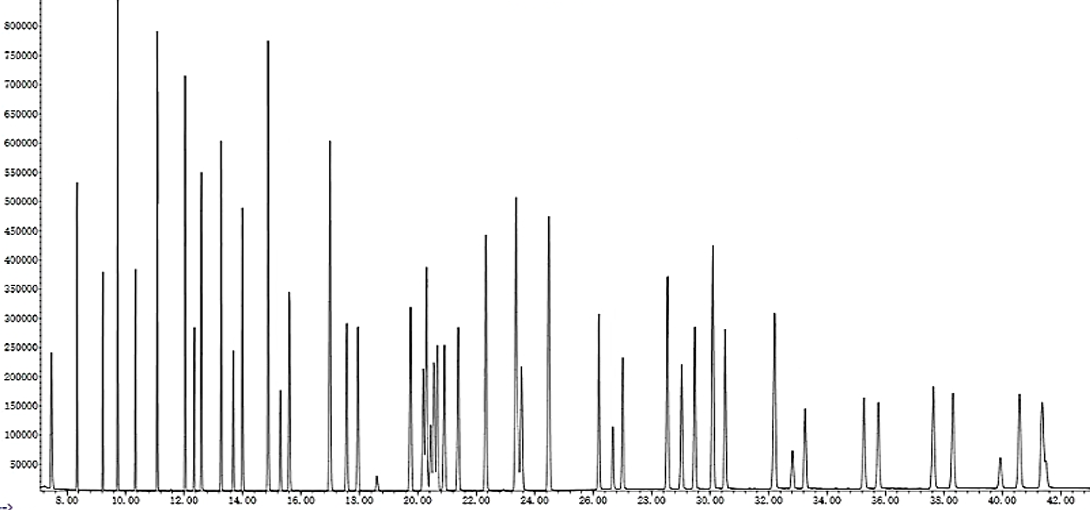


A


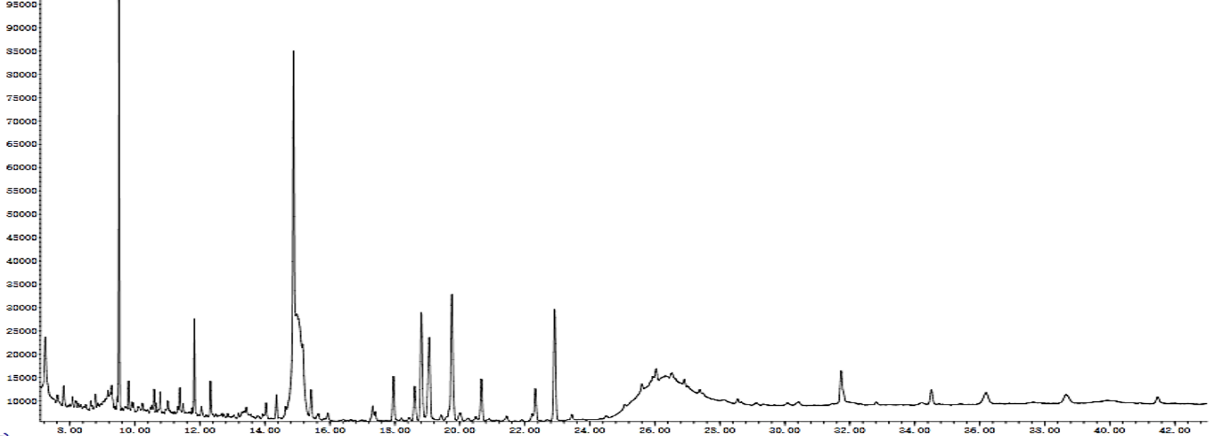


B


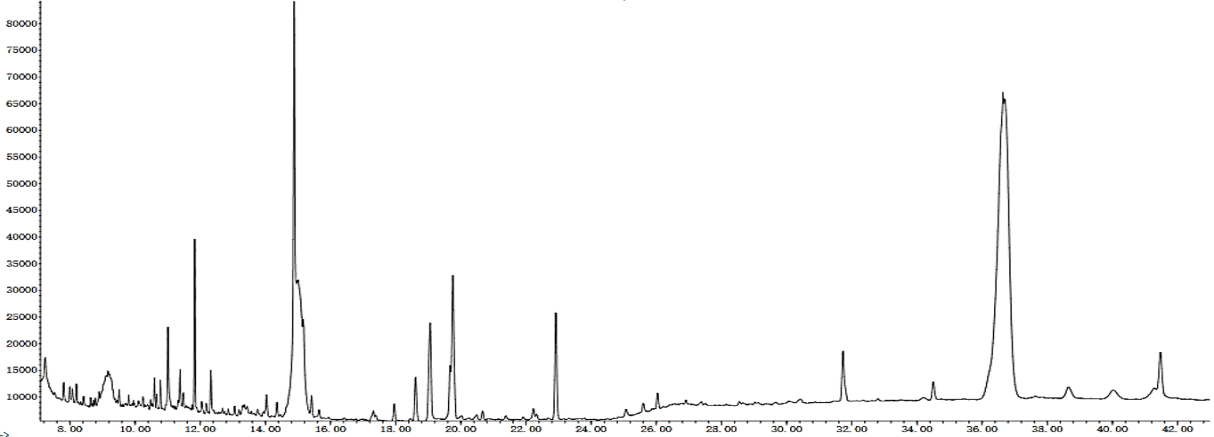


C


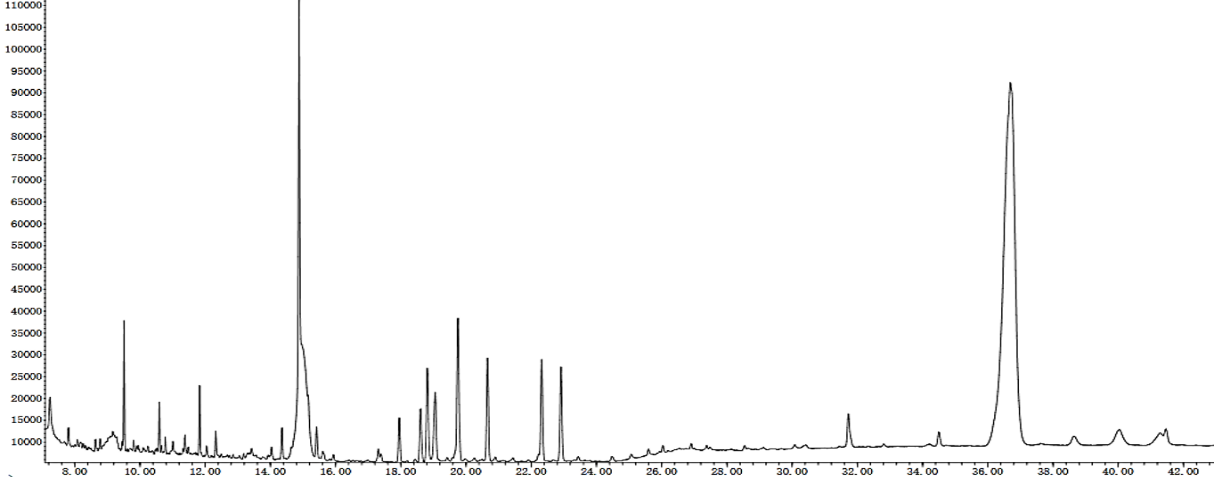


D

**Fig. S2 Total ion flow chromatograms of free fatty acids detected by GC-MS.** 49 free fatty acid standard mixtures (A), PC group (B), P group (C), C group (D). C represents the group of steam pot chicken soup.


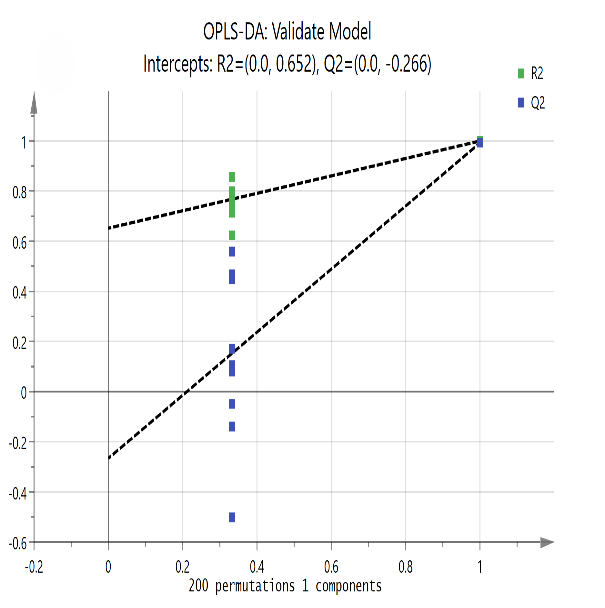

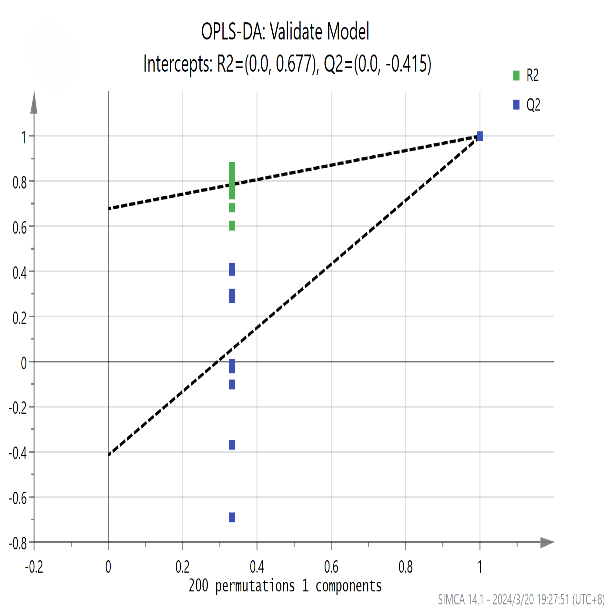


A B


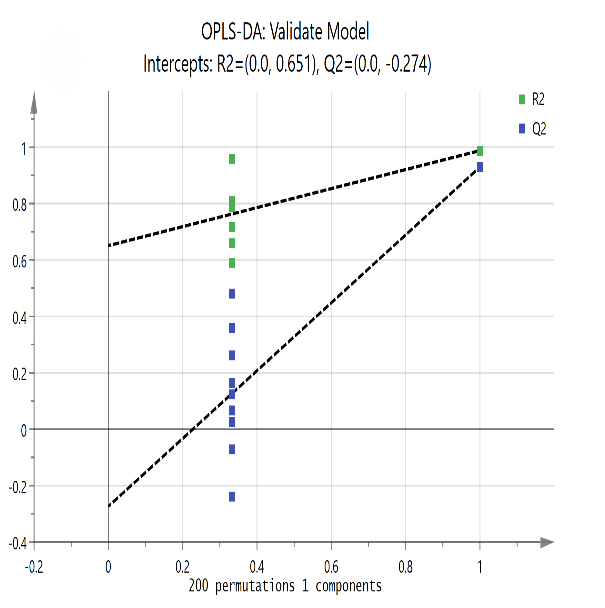

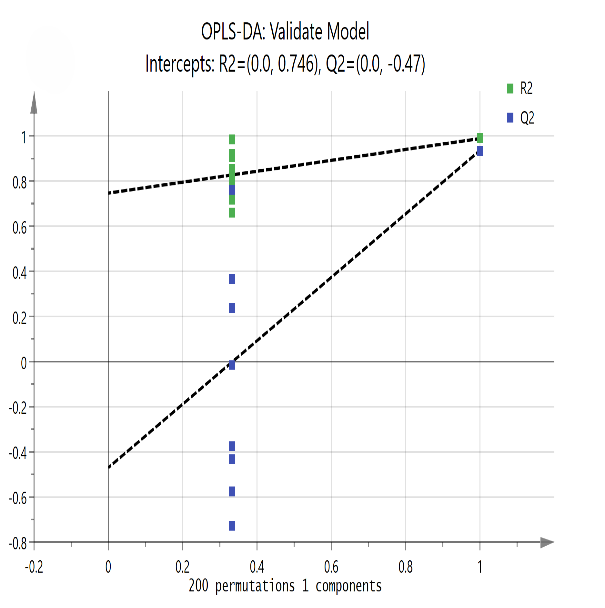


C D

**Fig. S3 Statistical analysis of differential small molecule metabolites between different groups.** OPLS-DA validation plots of PC vs. P group in ESI+ (A) and ESI- mode (B), OPLS-DA validation plots of PC vs. C group in ESI+ (C) and ESI- mode (D).


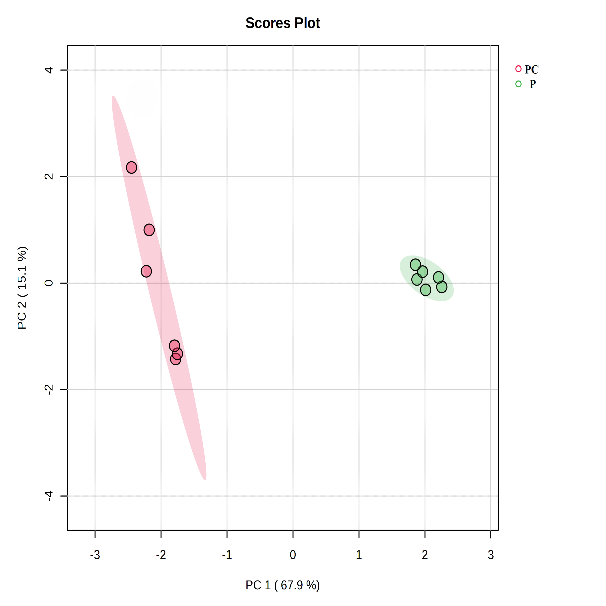

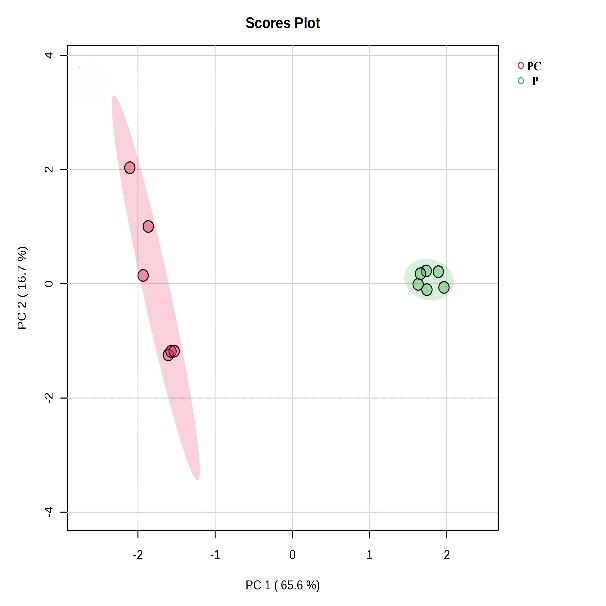


A B


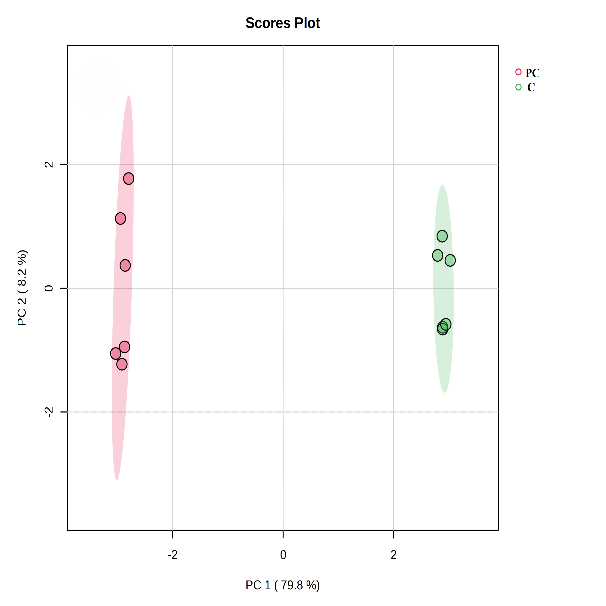

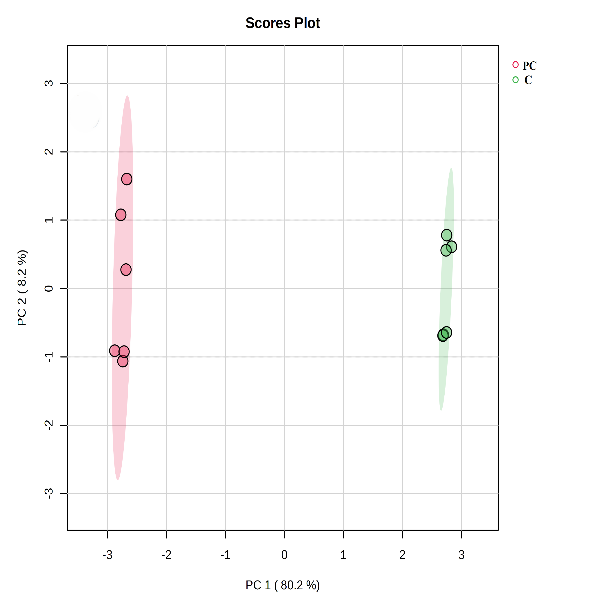


C D

**Fig. S4 Statistical analysis of differential small molecule metabolites between different groups.** PCA score plots of PC vs. P group in ESI+ (A) and ESI- mode (B), PCA score plots of PC vs. C group in ESI+ (C) and ESI- mode (D).


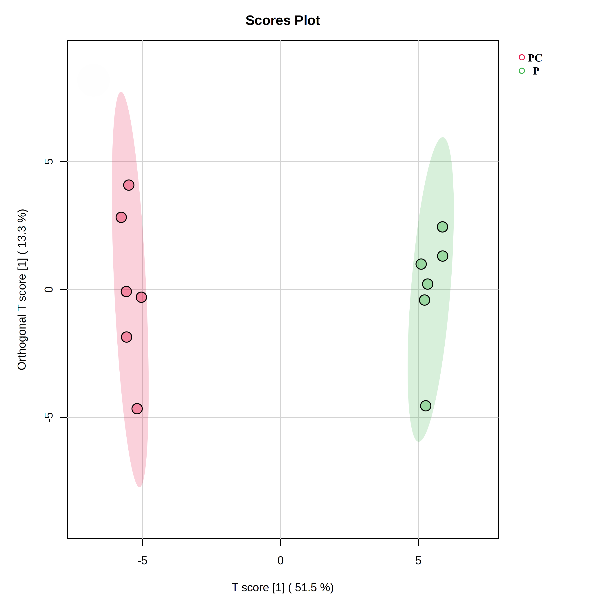

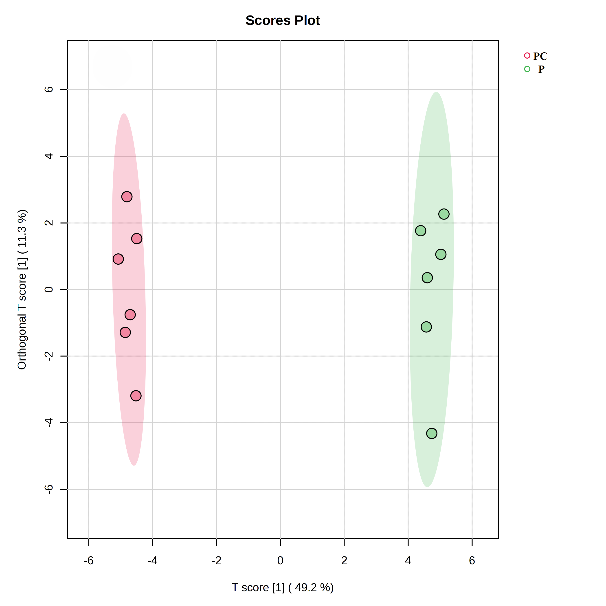


A B


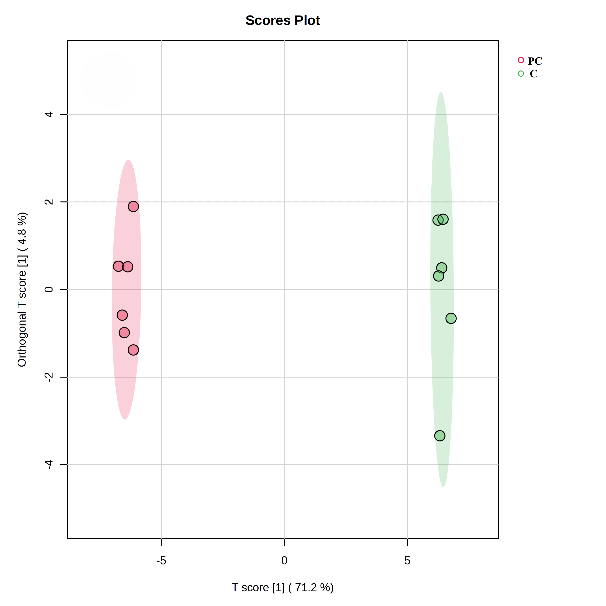

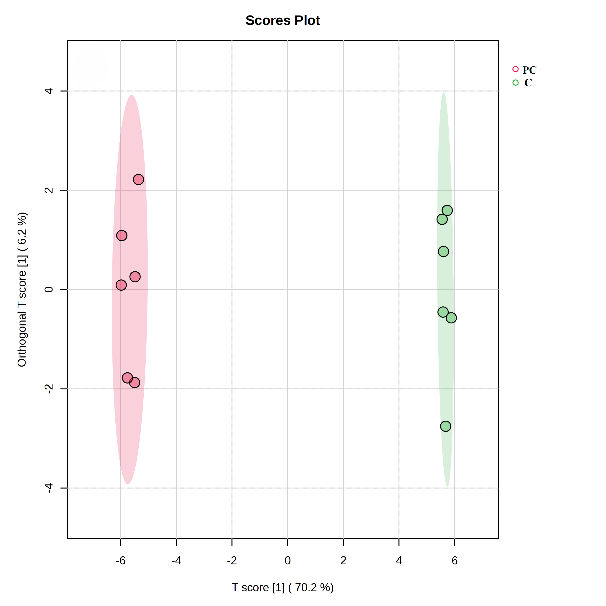


C D

**Fig. S5 Statistical analysis of differential small molecule metabolites between different groups.** OPLS-DA score of PC vs. P group in ESI+ (A) and ESI- mode (B), OPLS-DA score of PC vs. C group in ESI+ (C) and ESI- mode (D).

Table S1 HPLC determination results of saponin concentration (mg/L).

| Indicator ingredient name | PC | P |
| --- | --- | --- |
| R_1_ | 19.25±0.83^b^ | 24.90±0.41^a^ |
| R_g1_ | 61.04±1.50^b^ | 95.12±1.71^a^ |
| R_b1_ | 20.74±0.49^b^ | 35.13±0.49^a^ |

Note: Different letters on the shoulder of the same row indicate a significant difference (P<0.05).

Table S2 The top 15 differential metabolites of VIP values in ESI+ and ESI- mode.

| Mode | Name | Classification | VIP |
| --- | --- | --- | --- |
| POS | Asn-Gln | Amino acids and peptides | 2.5564 |
| POS | PC(16:0/18:1(9Z)) | Glycerophospholipids | 2.3986 |
| POS | 1-Oleoyl-2-palmitoyl-sn-glycero-3-phosphocholine | Lipids and lipid-like molecules | 2.3986 |
| POS | Pantetheine | Organic acids and their derivatives | 2.3902 |
| POS | Asparagine | Amino acids and peptides | 2.3656 |
| POS | Isoguanosine | Organic Heterocyclic Compounds | 2.3443 |
| POS | Ser-Gln | Amino acids and peptides | 2.1876 |
| POS | gamma-Glutamyl-beta-cyanoalanine γ | Organic acids and their derivatives | 2.1819 |
| POS | Leu-Lys | Organic acids and their derivatives | 2.1771 |
| POS | PC(35:4) | Glycerophospholipids | 2.1745 |
| POS | D-Fructose 6-phosphate | organic compound | 2.1667 |
| POS | 5-Guanidino-2-oxopentanoate | Organic acids and their derivatives | 2.1578 |
| POS | 1-Stearoyl-2-arachidonyl-sn-glycero-3-phosphocholine | Lipids and lipid-like molecules | 2.0729 |
| POS | PC(38:4) | Glycerophospholipids | 2.0729 |
| POS | LPC(16:0) | Glycerophospholipids | 2.0052 |
| NEG | 4-Guanidinobutanoate | Amino acids and peptides | 2.2948 |
| NEG | Pterine | Organic Heterocyclic Compounds | 2.2679 |
| NEG | Hydromorphone-3-.beta.-D-glucuronide | Alkaloids and derivatives | 2.2204 |
| NEG | 2-Ketocaproic acid | fatty acids | 2.1806 |
| NEG | Ketoleucine | fatty acids | 2.1806 |
| NEG | 3-Methyl-2-oxovaleric acid | Organic acids and their derivatives | 2.1806 |
| NEG | Undecanoic acid | fatty acids | 2.1569 |
| NEG | 9-Oxooctadecanoic acid | fatty acids | 2.0767 |
| NEG | cis-9,10-Epoxystearic acid | fatty acids | 2.0767 |
| NEG | O-Succinyl-L-homoserine | Organic acids and their derivatives | 2.0739 |
| NEG | D-Pinitol | organic compound | 1.9985 |
| NEG | Azelaic acid | fatty acids | 1.9969 |
| NEG | 3-Isobutylpentanedioic acid | Organic acids and their derivatives | 1.9969 |
| NEG | Pantothenic acid | fatty acids | 1.9292 |
| NEG | Glyceric acid | fatty acids | 1.9192 |
